# Relationship Between SES and Academic Achievement of Junior High School Students in China: The Mediating Effect of Self-Concept

**DOI:** 10.3389/fpsyg.2019.02513

**Published:** 2020-01-07

**Authors:** Shifeng Li, Qiongying Xu, Ruixue Xia

**Affiliations:** ^1^School of Psychology, Northwest Normal University, Lanzhou, China; ^2^Key Laboratory of Behavioral and Mental Health, Lanzhou, China

**Keywords:** socioeconomic status, self-concept, school academic achievement, adolescents, China

## Abstract

Over the past decades, the relationship between family socioeconomic status (SES) and academic achievement in school-age children has been well documented. However, the underlying mechanism of how family SES works on academic achievement remains unclear. In this study, we examine the possible role of self-concept in the relationship between SES and school academic achievement among 345 junior high school students in China. The results showed that both family SES and self-concept were significantly associated with the children’s Chinese and mathematics performance, and family SES was also significantly correlated with self-concept. The mediation analysis showed that self-concept partially mediated the relationship between SES and school academic achievement. These findings suggest that interventions targeting self-concept may be an effective way in which to improve children’s school academic achievement.

## Introduction

Socioeconomic status (SES), an index of one’s overall social status or prestige in society, is one of the most widely studied constructs in the social sciences. It is usually measured alongside education, occupational status, and income (Conger and Donnellan, 2007). Over the past decades, the relationship between SES and child development has been well documented (Bradley and Corwyn, 2002; Hackman et al., 2010; Aizer and Currie, 2014). Compared to children and adolescents growing up in families with high SES, those growing up in families with low SES demonstrated an increased health risk (Chen et al., 2002), higher rates of anxiety, depression, and conduct disorders (Wadsworth and Achenbach, 2005). Numerous studies also associated SES with the IQ level and academic achievement of children and adolescents (National Institute of Child Health and Human Development [Nicdh], 2005; White et al., 1993).

Over recent decades, the relationship between family SES and academic achievement in school-age children has been well documented across different sociocultural contexts. As early as 1966, the well-known Coleman Report revealed that family SES explained most variances in academic achievement (Coleman et al., 1966). Sirin (2005) conducted a comprehensive meta-analysis of 58 studies. For a sample from the United States, Sirin confirmed a medium to strong relation between SES and achievement with an average effect size of 0.27 (95% CI: 0.28–0.29). Similarly, Liu et al. (2019) conducted a meta-analysis based on 215,649 students from 78 independent samples. The sample from Mainland China demonstrated a moderate relation between SES and academic achievement (*r* = 0.243). However, the mechanism underlying the relationship between SES and child development remains unclear.

Several explanations have been proposed to interpret how family SES impacts child development. The most influential are the social causation model, social selection model, and sociocultural self model. The social causation model argues that social and economic conditions may influence children’s functioning and development (Conger et al., 2002). Some empirical studies supported this view, demonstrating that family economic hardship negatively affected parent emotion, relationship, and parenting behavior, which influence child development (Conger and Conger, 2002). Likewise, the investment of resources (including financial, social, and human capital) by families promotes the development of their children (Bradley et al., 2001). The social selection model takes a different approach to the relationship between SES and child development, arguing that individuals’ characteristics or attributes may influence their social and economic status (Mayer, 1997; Rowe and Rodgers, 1997). Some empirical research supports the social selection argument, showing that the positive characteristics of parents will reduce exposure to economic pressure in the family; decrease the likelihood of parents’ emotional, interparental, and parenting problems; and improve child well-being (Linver et al., 2002).

The sociocultural self model integrated and extended the key tenets of the social causation and social selection models (Stephens et al., 2012). It proposed that (1) social economic conditions and individual characteristics or attributes are interdependent forces that influence each other, and (2) both social economic conditions and individual characteristics or attributes indirectly influence individuals’ behavior through the self. In this model, self is defined as “a product of the ongoing mutual constitution of individuals and structures and serve to guide people’s behavior by systematically shaping how people construe situations” (Stephens et al., 2012, p. 733). Compatible with this view, recent studies indicated that academic self-efficacy mediated the relationship between SES and anticipated and actualized school performance (Wiederkehr et al., 2015). Interventions targeting the self and identity have been effective in reducing the racial/ethnic achievement gap for college (Cohen et al., 2006, 2009) and high school students in the United States (Sherman et al., 2013; Goyer et al., 2017).

Although there is evidence that improving family economic conditions reduces children’s risk of psychiatric disorders (Costello et al., 2003), and that interventions targeting individual attributes (e.g., attention) can significantly facilitate child development in low SES families (Neville et al., 2013), SES and individual characteristics remain relatively stable over a certain period. The sociocultural self model proposed a new and promising perspective in facilitating child development in families with low SES by changing the selves of students that emerged in a certain situation. However, previous studies on the mechanism of self mediating the relationship between SES and child development focused on western samples.

To our knowledge, no study has examined the role of self-concept in the relationship between SES and child development using a Chinese sample. It is well known that cultural experiences influence and determine one’s self. In China, under the influence of the Confucian culture, parents tend to pay much attention to their children’s learning activities and academic achievement. In most families, parents do their best to provide good learning conditions regardless of SES (Wong et al., 2012; Wang et al., 2014). Relevant research has shown that this type of parent support may influence the self-concept of children, which influences their school achievement (Xiao and Liu, 2017). Accordingly, some studies indicated the relation between good academic achievement and the praise and respect children received (Zhou et al., 2010). Whether this difference in cultural value influences the relationships between SES, self-concept, and school achievement remains unclear.

Therefore, in this study, we investigate the relationships between family SES, self-concept, and school academic achievement of Chinese junior school students in Mainland China. We hypothesized that family SES measured through parents’ education, occupation, and income would be significantly associated with children’s self-concept, which will influence their school achievement.

## Materials and Methods

### Participants

In total, 345 first-year students (age range = 9–17 years, *M*_age_ = 13.40 years, *SD* = 0.73; 52.4% female) at a junior middle school in Lanzhou, China were recruited as participants in this study. This school is a medium to large-sized community-based public elementary school with approximately 40–50 students in each class and approximately 3,000 students in total in grades 1–3. All students are native Mandarin speakers and native to Mainland China. We also followed ethics guidelines and obtained permission from the school principals, teachers, parents, and children. Consent was first obtained from the school principals and teachers. Then, parents indicated their consent by signing a form distributed at a parent meeting or brought home by their children.

### Measures and Procedure

All children completed a demographic question (age, gender, parents’ level of education, parents’ occupational status or what jobs the parents held, and annual household income) and a self-concept scale in the middle of the second semester. Children completed the demographic questions by taking the questionnaire home and consulting with their parents. The children were asked to complete the self-concept scale by themselves. At the end of the semester, we obtained their final exam scores for two subjects (Chinese and mathematics) as indicators of their school academic achievement.

#### SES

Although there is no consensus on how to measure SES, it is agreed that a stable measure thereof should incorporate education, occupation, and income (Bradley and Corwyn, 2002). Therefore, in this study, we used parents’ level of education, occupation, and annual household income to estimate the children’s family SES. Parents’ level of education was measured on a seven-point Likert scale: 1 = primary grade 3 or below, 2 = primary grade 4 to 6, 3 = middle school, 4 = high school, 5 = 3 year college, 6 = 4-year university, 7 = postgraduate. Parents’ occupation was measured using the Occupational Prestige Scale (Li, 2005), in which 81 occupations are rated and their scores standardized as 0–100. A higher score represents the higher prestige of that occupation. Annual household income was measured on a ten-point Likert scale: 1 = less than 10,000; 2 = between 10,000 and 30,000; 3 = between 30,000 and 50,000; 4 = between 50,000 and 100,000; 5 = between 100,000 and 150,000; 6 = between 150,000 and 200,000; 7 = between 200,000 and 300,000; 8 = between 300,000 and 500,000; 9 = between 500,000 and 1,000,000; 10 = more than 1,000,000 Chinese Yuan per year.

#### Self-Concept

Children’s self-concept was assessed using the Children and Adolescents Self-Recognition Scale (CASRS) developed by Dong and Lin (2011). This scale includes 18 items, which assess the children’s perceived self through their past experience and understanding of this past experience (Shavelson et al., 1976). Sample items are: “Most of my courses are very good,” “I did well in most of my courses.” Children were asked to rate all items on a four-point Likert scale (1 = strongly disagree, 4 = strongly agree). The sum of the scores of each item was the final score of this scale, with higher scores indicating a more positive perceived self-concept. Previous studies confirmed the high validity and reliability of the CASRS (Dong and Lin, 2011). In this study, the internal consistency reliability coefficient (Cronbach’s α) of the scale was 0.82.

#### School Academic Achievement

School academic achievement in this study was defined as the children’s performance in school subject areas such as language literacy and mathematics. Because there are no standardized language and mathematics tests in China, following previous studies (Donnelly et al., 2016; Xiang et al., 2017), we collected the children’s final exam scores for two subjects (Chinese and mathematics) as indicators of their school academic achievement. In China, school achievement is usually assessed through a Teacher-Edited Test, which examines students’ learning and understanding in school subject areas such as mathematics and language literacy in the middle and at the end of each semester. In this study, the test raw score for each subject area ranged from 0–150, with higher scores indicating higher performance in that subject area.

### Data Analysis

First, descriptive statistics (mean scores, standard deviations, and range) and Pearson’s correlations were calculated using SPSS 22.0 for each variable. Then, the mediation model was tested in Mplus 7.0 (Muthen and Muthen, 1998/2012). In mediation model, we use latent construct to estimate the SES with five observed variables (father’ level of education, mother’ level of education, father’s occupational prestige, mother’s occupational prestige, and annual household income).

## Results

### Descriptive Statistics, Internal Reliability, and Inter-Correlations

Table 1 shows the means, standard deviations, and ranges of all measures in this study. Table 2 shows the inter-correlations for all measures controlling for sex and age. As Table 2 shows, most SES measures (parents’ level of education, occupation, and annual household income) were significantly correlated with the self-concept as well as performance in Chinese and mathematics, respectively. In addition, self-concept was also significantly correlated with performance in Chinese and mathematics.

**TABLE 1:** Means, standard deviations, range, and reliability for all measures (*n* = 345).

**Variable**	***M***	***SD***	**Range**	**Cronbach’s**
Mother’s education level	3.40	1.22	1–7	–
Father’s education level	3.30	1.47	1–7	–
Mother’s occupational prestige	38.83	13.43	9.73–87.92	–
Father’s occupational prestige	46.92	12.95	26.35–87.92	–
Annual household income level	3.80	1.81	1–10	–
Self-concept	39.83	5.05	20–53	0.82
Chinese achievement	79.26	10.84	31–121	–
Mathematics achievement	76.89	19.55	12–141	–

**TABLE 2:** Correlations among SES measures, self-concept, Chinese, and mathematics performance controlling for sex and age.

**Variable**	**1**	**2**	**3**	**4**	**5**	**6**	**7**	**8**
1. Mother’s education level	1.00							
2. Father’s education level	0.47^∗∗∗^	1.00						
3. Mother’s occupational prestige	0.33^∗∗∗^	0.14^∗^	1.00					
4. Father’s occupational prestige	0.29^∗∗∗^	0.25^∗∗∗^	0.51^∗∗∗^	1.00				
5. Annual household income level	0.16^∗^	0.21^∗∗∗^	0.21^∗∗∗^	0.21^∗∗∗^	1.00			
6. Self-concept	0.12^∗^	0.16^∗∗^	0.12^∗^	0.04	0.20^∗∗∗^	1.00		
7. Chinese performance	0.22^∗∗∗^	0.19^∗∗∗^	0.08	0.01	0.14^∗^	0.18^∗∗^	1.00	
8. Mathematics performance	0.16^∗∗^	0.08	0.16^∗∗^	0.05	0.16^∗∗^	0.23^∗∗∗^	0.63^∗∗∗^	1.00

**n* = 345; ^∗^*P* < 0.05; ^∗∗^*P* < 0.01; and ^∗∗∗^*P* < 0.001.*

### Mediation Model

To examine the possibility that the relationship between SES and school academic achievement was mediated by self-concept, we conducted two mediation analyses. The first analysis tested the hypothesis that self-concept mediated the relationship between SES and performance in Chinese. The second tested the hypothesis that self-concept mediated the relationship between SES and performance in mathematics.

#### Performance in Chinese

**FIGURE 1:**
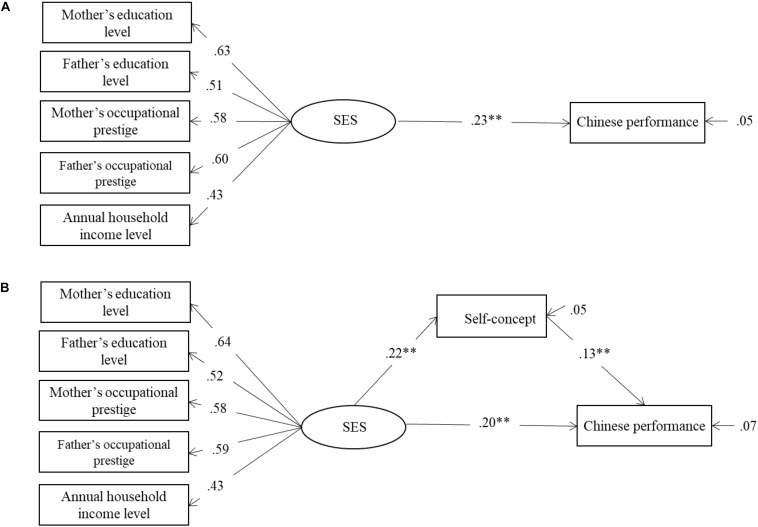
**(A)** The relationship between SES and Chinese performance. **(B)** Self-concept mediate the relationship between SES and Chinese performance. SES = socioeconomic status. *^∗^p* < 0.01; ^∗∗∗^*p* < 0.001; *n* = 345.

Figure 1 shows the results for performance in Chinese. As shown in Figure 1A, first, a direct model was used to test the relationship between SES and school Chinese achievement. As expected, the direct effect (β = 0.23, *p* < 0.01) from SES to school Chinese achievement was significant. Then, the mediation model was used to examine the potential mediating effects of self-concept on the relationship between SES and school Chinese achievement. As shown in Figure 1B, when self-concept entered as a mediator, the direct effect (β = 0.20, *p* = 0.01) from SES to school Chinese achievement was still statistically significant, and the indirect effect of self-concept was also significant (β = 0.027, *p* < 0.05). These results confirmed that self-concept partially mediated the relationship between SES and Chinese achievement.

#### Performance in Mathematics

**FIGURE 2:**
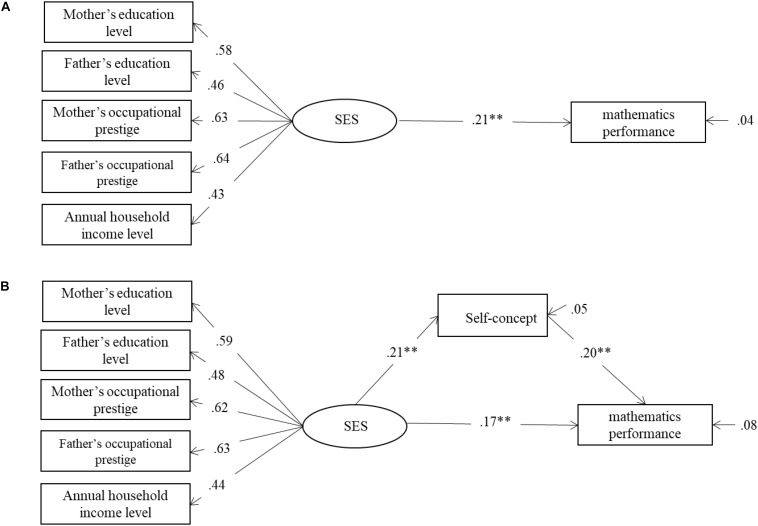
**(A)** The relationship between SES and mathematics performance. **(B)** Self-concept mediate the relationship between SES and mathematics performance. SES = socioeconomic status. *^∗∗^p* < 0.01; ^∗∗∗^*p* < 0.001; *n* = 345.

The same analyses were repeated for performance in mathematics. As shown in Figure 2A, the direct model showed that the direct effect (β = 0.20, *p* < 0.01) from SES to school mathematics achievement was significant. The mediation model showed that when self-concept entered as a mediator (Figure 2B), the direct effect (β = 0.17, *p* < 0.01) from SES to school mathematics achievement was still statistically significant, and likewise, the indirect effect of self-concept was also significant (β = 0.042, *p* < 0.05).

### Moderation Model

The possible moderating effects of self-concept in the relationship between SES and academic achievement (in both Chinese and mathematics) were examined using hierarchical regression analyses. The interactions of SES with self-concept were represented by multiplying the standard SES score with the standard score of the self-concept measures. However, these interactions demonstrated no significant effects after sex, age, SES, and self-concept were put into previous blocks in the corresponding models (all *p* > 0.05).

## Discussion

Social inequalities have profound effects on the development of children and adolescents. Reducing social class disparities in their development is on the global research agenda (Adler et al., 1993; Cohen et al., 2006; Stephens et al., 2012). However, the mechanism underlying the relationship between SES and child development remains unclear. This study was designed to explore the possible mediating role of self-concept in the relationship between SES and academic achievement among junior high school students in China. We found a moderate relation between SES and academic achievement (Chinese: *r* = 0.18; mathematics: *r* = 0.23). This finding is aligned with that of Liu et al. (2019), which reported that the overall relationship between Chinese students’ SES and academic achievement was moderate (*r* = 0.24). However, our finding differs from that of the PISA report (*r*^2^ = 0.18 is about *r* = 0.42) in the B-S-J-G area (Beijing-Shanghai-Jiangsu-Guangdong) of China (OECD, 2016). One reason for the differences in the results of studies may be related to the level of socio-economic and cultural development of the selected sample. B-S-J-G are among the top four provinces in China in terms of economics, residents’ incomes, and school education, whereas Gansu (in the current study) is among the bottom provinces in terms of these aspects (NBSC, 2016). Compatible with this explanation, relevant studies showed that the relationship between SES and academic achievement in developing countries, especially in low-income countries, was weaker than that in developed countries (Heyneman and Loxley, 1983; OECD, 2016). Further studies are needed to examine how the levels of socio-economic and cultural development modulate the relationship between SES and academic achievement in different provinces or regions of China.

One important implication of this study is that SES predicted academic achievement partially through the mediating effect of self-concept. This is generally consistent with previous studies using a western sample, which revealed that self-efficacy mediated the relationship between SES and anticipated and actualized school performance (Wiederkehr et al., 2015). The overall findings support the theoretical framework that children’s academic achievement is indirectly associated with SES through the mediating effect of self-concept across cultures. These results suggest that families with a high SES should help children form and sustain a positive self-concept, which is associated with better academic school performance. Indeed, research has documented that children from families with low SES usually experience more economic hardship, a lack of various resources, and higher threats to social identity such as negative stereotypes regarding their intellectual ability (Croizet and Claire, 1998) and social belonging (Veland et al., 2009). These disadvantaged economic and psychological conditions may pose a chronic threat to children’s self-integrity, undermining their academic performance (Cohen et al., 2006; Walton and Cohen, 2011). These results were also consistent with the view of the sociocultural self model, namely that socioeconomic conditions influence individuals’ behavior through how they define themselves in a certain situation (Stephens et al., 2012).

In addition to the significant indirect effect of family SES on school achievement through self-concept, we also found that family SES directly affects school achievement. This implies that self-concept may not completely explain the relationship between family SES and school achievement. This finding differed somewhat from that of a study by Wiederkehr et al. (2015) on French children that identified the fully mediating role of self-concept in the relationship between family SES and school performance. Differences in other important factors closely related to family SES such as learning materials available in the home and parents’ stimulation of their children to learn may also play important roles in the link between family SES and the school achievement of Chinese children. According to the family investment model, parents’ material and interpersonal investment in their children may at least partially explain the association between family SES and children’s development (Kalil and Deleire, 2004; Conger and Donnellan, 2007). Nevertheless, the results of this study demonstrated the important role of self-concept in the relationship between family SES and the academic achievement of Chinese children.

The findings of this study have important implications for possible interventions to improve academic school achievement. Although SES is a relatively stable condition and difficult to change, our results suggest that helping children form and sustain a positive self-concept may improve their academic school achievement and reduce social inequalities in child development. Accordingly, recent studies based on the sociocultural self model showed that interventions targeting the self and identity were effective in reducing the racial/ethnic achievement gap for college and high school students in the United States (Cohen et al., 2006, 2009; Sherman et al., 2013; Goyer et al., 2017). Further studies are needed to examine whether these interventions are also an effective way to reduce the social class (SES) disparities in academic achievement in China.

Finally, some limitations of this study should be acknowledged. First, convenience sampling may hinder the generalizability of the results. Second, the cross-sectional design of the study may have caused difficulties in establishing the causal relationships between variables. Previous studies showed a reciprocal relationship between self-concept and academic achievement (Marsh et al., 1999; Marsh and O’Mara, 2008). Longitudinal studies are needed to examine the dynamic relationship between SES, self-concept, and school academic achievement.

In summary, the findings of this study confirmed the role of family SES in academic achievement among junior high school students in northwest China. Furthermore, the current study also extended the extant literature by demonstrating that family SES influences children’s academic school achievement partly through their self-concept in China. These findings suggested that disadvantageous family backgrounds may have a negative impact on how children defined themselves in school situation, and ultimately influence on their academic school achievement. Intervention target on help low SES students to maintain self-integrity may an effective way in reducing the social stand achievement gap for middle school students in China.

## Data Availability Statement

The datasets generated and analyzed during this study are available from the corresponding author on reasonable request.

## Ethics Statement

The studies involving human participants were reviewed and approved by the Ethics Committee of the School of Psychology, NWNU. Written informed consent to participate in this study was provided by the participants’ legal guardian/next of kin.

## Author Contributions

SL and RX conceived the research. QX participated in performing the research. All authors participated in writing the manuscript.

## Conflict of Interest

The authors declare that the research was conducted in the absence of any commercial or financial relationships that could be construed as a potential conflict of interest.
